# Immediate Latissimus Dorsi Flap Reconstruction: Assessing Aesthetic Outcomes Following Mastectomy in Breast Cancer Patients

**DOI:** 10.7759/cureus.64874

**Published:** 2024-07-18

**Authors:** Vekhotso Nyekha, Meghraj Kundan, Vivek Belsariya, Soorya VK, Ayush Agarwal

**Affiliations:** 1 General Surgery, Vardhman Mahavir Medical College and Safdarjung Hospital, New Delhi, IND; 2 General Surgery, King George's Medical University, Lucknow, IND

**Keywords:** breast-q score, early breast cancer, aesthetic approach, immediate reconstruction, cosmetic outcomes, quality of life (qol), breast cancer outcomes, skin-sparing mastectomy, autologous flap reconstruction, latissimus dorsi

## Abstract

Background: Breast Cancer has now become the leading cause of cancer-related deaths among women. In a traditional radical mastectomy, there can be complications that may affect the physiological characteristics of the breast and subsequently cause profound psychological stress to the patients. Hence, latissimus dorsi (LD) flap reconstruction provides an aesthetic approach in patients undergoing mastectomy. The goal is to maximize the flap's soft tissue coverage while minimizing the magnitude of donor site defect and complication.

Methods: A prospective observational study was conducted in the Department of General Surgery, Safdarjung Hospital, New Delhi, India, where 30 breast cancer patients were enrolled and had undergone mastectomy with immediate LD flap reconstruction. Cosmetic assessments using BREAST-Q questionnaires were conducted postoperatively at various intervals starting from postoperative day one, week two, and week six. The subjective evaluation was done by the patient, while a blinded nurse and surgeon did the objective assessment.

Result: The majority (n=23, 76.7%) were aged 31-50 years. Initial postoperative BREAST-Q scores declined but significantly improved by week six, attributed to gradual wound healing over time, resulting in improved breast shape and contour. The objective scoring done by the blinded surgeon and nurse improved at six weeks compared to two weeks postoperatively. Almost similar outcomes were observed between preoperative and six-week postoperative scores with a significant overall p-value of <0.001. No significant statistical differences were noted between blinded surgeons and nurses for objective scoring.

Conclusion: The rising trend of breast cancer in younger demographics emphasizes the importance of balancing cosmetic satisfaction with oncological outcomes. Immediate LD flap breast reconstruction provides a reliable means for soft tissue coverage with acceptable perioperative morbidities for patients undergoing mastectomy. Complication rates were acceptable, with donor site seroma, surgical site infection (SSI), and shoulder weakness among them. They could be prevented or treated (prolonged drain in situ, quilting sutures, and seroma aspiration) or resolved with time (SSI and shoulder function).

## Introduction

The most common cancer according to global statistics for women is breast carcinoma, which accounts for 25.1 percent of all cancers [1]. India is also following the same trend with the most common cancer among women being breast cancer, accounting for 28.8 percent [2]. Many young women with breast cancer expect the treatment to result in long-term survival as well as positive cosmetic and psychological outcomes [3]. Traditional radical mastectomy can impact the physiological characteristics and the unique curvilinear beauty of patients and can cause serious psychological stress in patients [4]. Breast-conserving surgery (BCS) and other reconstructive surgeries help patients save their breasts, maintain their image, and meet their psychological demands while also keeping in mind the oncological outcomes [5].

The latissimus dorsi (LD) flap is the first-line option in autogenous breast cancer reconstruction for patients who are not candidates for the transverse rectus abdominis muscle (TRAM) flap. It can provide tissue to correct partial mastectomy or lumpectomy defects, augment thin or unreliable skin flaps over an implant, or maximize the aesthetic outcome of a prophylactic mastectomy [6]. The LD flap pedicle is very reliable and consistent, which makes it a reproducible surgical technique with avoidance of microvascular anastomoses. In addition to this, this muscle can take various shapes and orientations, which has rendered this flap well-recognized [7]. During the last decade, the concept of LD flap surgery has greatly refined reconstructive breast surgery. Its goal is to maximize the soft tissue coverage provided by the flap while minimizing the magnitude of donor site defects and complications, ensuring positive aesthetic and oncological outcomes.

## Materials and methods

A prospective observational study was conducted between July 2022 to December 2023 in Safdarjung Hospital, New Delhi, India. We enrolled 30 breast cancer patients undergoing mastectomy with immediate LD flap reconstruction. Institutional Review Board and Ethics Committee of Vardhman Mahavir Medical College and Safdarjung Hospital, New Delhi, India, reviewed and approved this study (approval number: IEC/VMMC/SJH/Thesis/06/2022/CC-133). All patients of breast cancer undergoing mastectomy were included in the study while those patients who had previous ipsilateral LD flap reconstruction done or attempted were excluded. Patients underwent detailed preoperative workup in the ward, including triple assessment and other investigations for details of tumor staging of the disease, as per the eighth edition American Joint Committee on Cancer (AJCC) guidelines. Complete blood counts and other investigations regarding fitness for surgery were performed, and all patients underwent digital photography from a fixed distance of 60 cm of the profile of the bilateral breasts, after we obtained informed consent, so that we could have a baseline profile. The same surgical team performed the tumor resection, donor flap selection, and oncoplastic breast reconstruction in the same elective settings.

Cosmetic assessments were conducted at postoperative day one, at two weeks, and at six weeks, with subjective evaluation by patients themselves and objective assessments by the blinded nurse and blinded surgeon using standardized BREAST-Q questionnaires. The conceptual framework of the BREAST-Q module comprised two overarching themes, which were patient satisfaction and quality of life. The patients were assessed based on their physical, psychosocial, and sexual well-being. These self-assessments by the patients were concerned with the satisfaction with their breasts following surgery, how comfortable they were wearing a brassiere, and how it had affected their social and sexual lives. The primary objective was to assess the aesthetic outcomes following mastectomy with immediate latissimus dorsi flap reconstruction using BREAST-Q score, while secondary objectives included assessing flap-related complications like flap necrosis, seroma, and infection. 

Breast-Q is a validated, multidimensional questionnaire-based tool for assessing patient-reported outcome measures (PROMs) after breast reconstruction. It measures a patient's experience and quality of life through a hierarchy of questions that examine physical, psychological, and sexual well-being, cosmetological experiences, and overall satisfaction. The results were analyzed using the Q-Score software (Rasch Unidimensional Measurement Model Laboratory, Perth, Australia) with an average score of 100 for the four domains of breast appearance, emotional, and sexual well-being. Responses were compared using the chi-square analysis for categorical responses and the unpaired student's t-test. Statistical outcomes were analyzed by a licensed version of IBM SPSS Statistics for Windows, Version 21, (Released 2012; IBM Corp., Armonk, New York, United States).

## Results

Thirty breast cancer patients underwent mastectomy with immediate LD flap reconstruction. Preoperative and postoperative subjective and objective assessments were conducted using BREAST-Q score on postoperative day (POD)1, at two weeks postoperation, and at six weeks postoperation. The results are presented below.

A total of 20.0% (n=six) of the participants were 21-30 years old. Most of the participants (n=14, 46.7%) were between the ages of 31 and 40 years. A total of 30.0% (n=nine) of the participants were 41-50 years old. Only one participant (3.3%) was aged between 51 and 60 years (Table 1). 

**Table 1:** Age distribution of the patients

Age	Frequency	Percentage	95% CI
21-30 Years	6	20.0%	8.4% - 39.1%
31-40 Years	14	46.7%	28.8% - 65.4%
41-50 Years	9	30.0%	15.4% - 49.6%
51-60 Years	1	3.3%	0.2% - 19.1%

Non-parametric tests (Friedman test) were used to explore whether the BREAST-Q changed significantly over time. The mean BREAST-Q decreased from a maximum of 83.33 at the preoperative time point to a minimum of 68.80 at the POD1 time point and then increased to 79.20 at the week six time point. This change was statistically significant (Friedman test: χ2 = 83.2, p = <0.001) (Table 2). 

**Table 2:** Assessment of the changes in BREAST-Q over time *p-value <0.05 is taken as significant SD: standard deviation; IQR: interquartile range

Time point	BREAST-Q			Friedman test	
	Mean (SD)	Median (IQR)	Range	χ2	p-value
Preoperative	83.33 (3.76)	82.00 (6.00)	76.00 - 90.00	83.2	<0.001*
postoperative day (POD)1	68.80 (2.81)	70.00 (4.00)	64.00 - 76.00		
Week two	69.40 (3.61)	70.00 (4.00)	62.00 - 78.00		
Week six	79.20 (4.51)	78.00 (8.00)	72.00 - 88.00		

Post-hoc pairwise analysis was performed to explore at which time points the BREAST-Q score differed significantly from the preoperative time point. The BREAST-Q score differed significantly from the preoperative time point at the following time points: POD1 and week 2. The maximum change from the preoperative time point was observed at the POD1 time (Table 3).

**Table 3:** Comparison of BREAST-Q at various postoperative time points vs. the preoperative time point *p-value <0.05 is taken as significant. Post-hoc pairwise tests (Friedman test) performed using the Nemenyi test method for p-value correction. SD: standard deviation; IQR: interquartile range; POD1: postoperative day one

Time point	Mean (SD) of difference	Median (IQR) of difference	Range of difference	p-value
POD1	-14.53 (3.96)	-14.00 (4.00)	-24.00 - -6.00	<0.001*
Week two - postoperative	-13.93 (3.91)	-15.00 (5.50)	-20.00 - -6.00	<0.001*
Week six - postoperative	-4.13 (1.81)	-4.00 (1.50)	-8.00 - -2.00	0.014*

Non-parametric tests (Friedman test) were used to explore whether the blind surgeon score changed significantly over time. The mean blind surgeon score increased from a minimum of 16.00 at the POD1 time point to a maximum of 21.87 at the week six time point. This change was statistically significant (Friedman test: χ2 = 56.4, p = <0.001) (Table 4). 

**Table 4:** Assessment of changes in blinded surgeon score over time *p-value <0.05 is taken as significant. SD: standard deviation; IQR: interquartile range

Time point	Blind surgeon score			Friedman test	
	Mean (SD)	Median (IQR)	Range	χ2	p-value
Postoperative day (POD)1	16.00 (1.36)	16.00 (2.00)	14.00 - 18.00	56.4	<0.001*
Week two	17.07 (1.17)	17.00 (2.00)	14.00 - 20.00		
Week six	21.87 (1.66)	22.00 (2.75)	19.00 - 25.00		

Post-hoc pairwise analysis was performed to explore at which time points the blind surgeon score differed significantly from the POD1 time point. The blind surgeon score differed significantly from the POD1 timepoint at the following time points: week two and week six. The maximum change from the POD1 time point was observed at the week six timepoint (Table 5).

**Table 5:** Comparision of blind surgeon scores at various time points vs. postoperative day (POD)1 *p-value <0.05 is taken as significant. Post-hoc pairwise tests (Friedman test) performed using the Nemenyi test method for p-value correction. SD: standard deviation; IQR: interquartile range

Time point	Mean (SD) of difference	Median (IQR) of difference	Range of difference	p-value
Week two - postoperative	1.07 (0.87)	1.00 (2.00)	0.00 - 2.00	0.027*
Week six - postoperative	5.87 (1.22)	6.00 (2.00)	4.00 - 9.00	<0.001*

Non-parametric tests (Friedman test) were used to explore whether the blind nurse score changed significantly over time. The mean blind nurse score increased from a minimum of 15.73 at the POD1 time point to a maximum of 22.03 at the week six time point. This change was statistically significant (Friedman test: χ2 = 55.4, p = <0.001) (Table 6). 

**Table 6:** Assessment of changes in the blind nurse score over time *p-value <0.05 is taken as significant. SD: standard deviation; IQR: interquartile range

Time point	Blind nurse score			Friedman test	
	Mean (SD)	Median (IQR)	Range	χ2	p-value
Postoperative (POD)1	15.73 (1.14)	16.00 (1.00)	14.00 - 18.00	55.4	<0.001*
Week two	16.63 (1.33)	17.00 (2.00)	14.00 - 19.00		
Week six	22.03 (1.54)	22.00 (2.00)	20.00 - 25.00		

Post-hoc pairwise analysis was performed to explore at which time points the blind nurse score differed significantly from the POD1 time point. The blind nurse score differed significantly from the POD1 timepoint at the following time points: week two and week six. The maximum change from the POD1 time point was observed at the week six time point (Table 7).

**Table 7:** Comparision of blind nurse scores at various time points vs. postoperative day (POD)1 time point *p-value <0.05 is taken as significant. Post-hoc pairwise tests (Friedman test) performed using the Nemenyi test method for p-value correction. SD: standard deviation; IQR: interquartile range

Time point	Mean (SD) of difference	Median (IQR) of difference	Range of difference	p-value
Week two - postoperative	0.90 (0.84)	1.00 (1.00)	-1.00 - 3.00	0.027*
Week six - postoperative	6.30 (1.26)	6.00 (1.00)	3.00 - 9.00	<0.001*

The postoperative complication rates in terms of seroma formation, surgical site infection (SSI), and flap necrosis as tabulated in Table 8 showed that five (16.7%) of the participants developed seroma and none had flap necrosis. About 13.3% (n=four) had SSI post surgery.

**Table 8:** Summary of complications

Complication	Yes	No
Seroma	5 (16.7%)	25 (83.3%)
Flap necrosis	0 (0.0%)	30 (100.0%)
surgical site infection (SSI)	4 (13.3%)	26 (86.7%)

**Figure 1:**
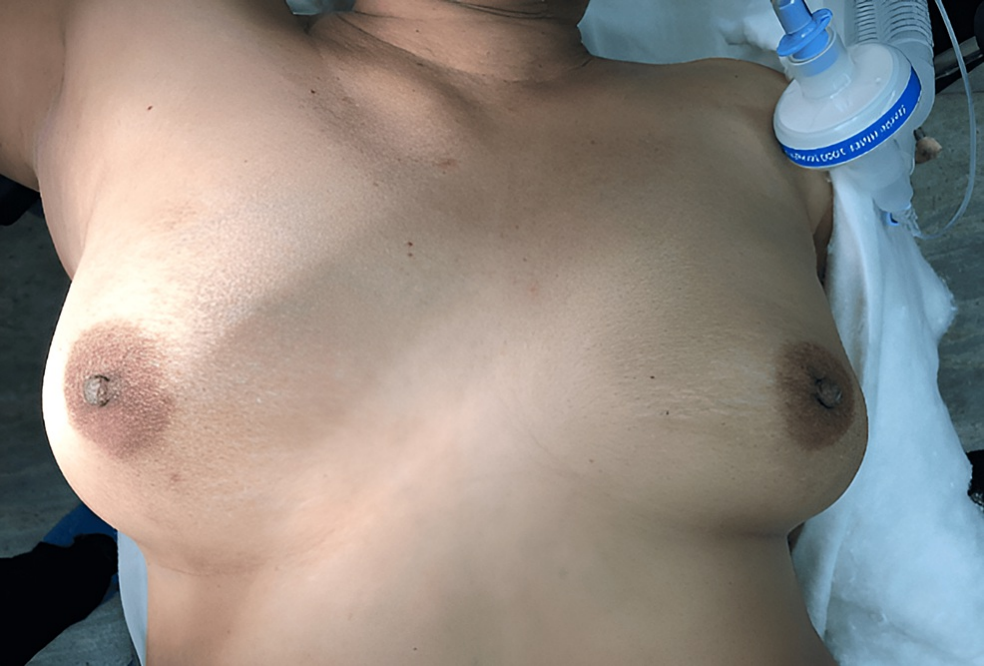
Preoperative image showing bilateral breasts

Below are images depicting cosmetic results over time following LD flap reconstruction starting from the preoperative time point to six weeks postoperation. Figure 1 shows a preoperative bilateral breast of the patient immediately before surgery.

**Figure 2:**
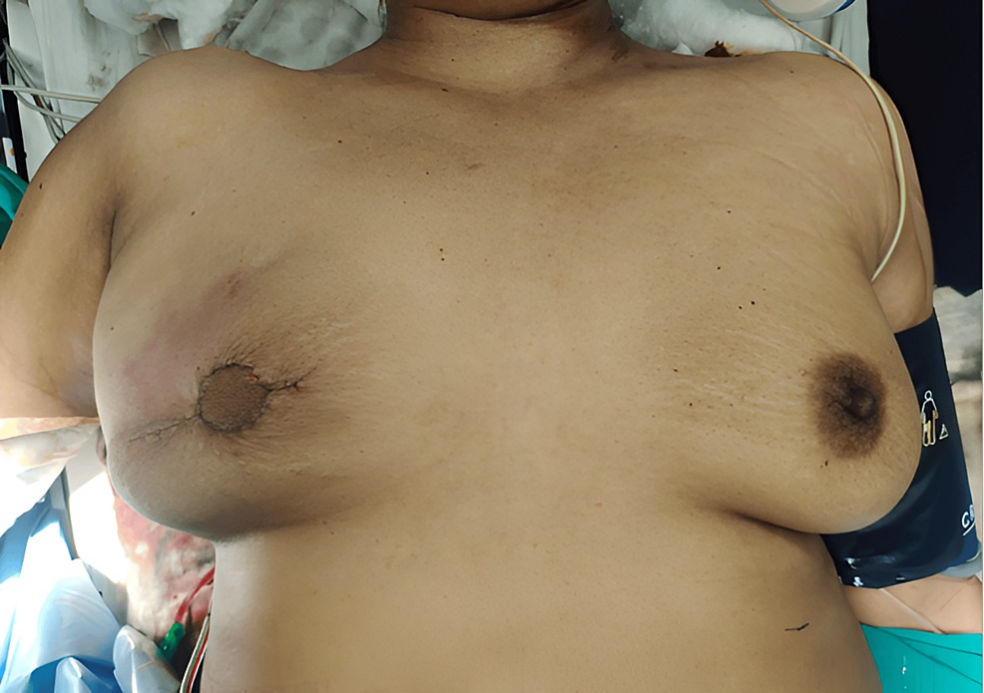
Immediate postoperation image following LD flap reconstruction

All patients underwent mastectomy (skin-sparing) with axillary clearance followed by immediate LD flap reconstruction in the same surgical setting (Figure 2).

**Figure 3:**
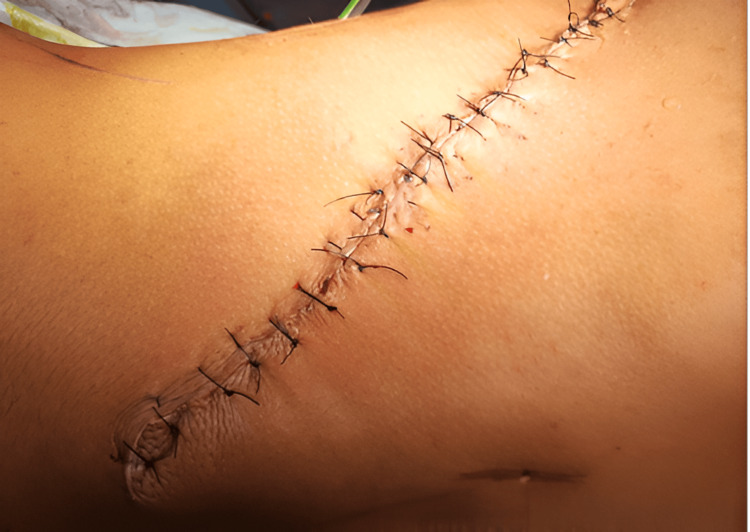
Postoperative donor site

Figure 3 shows the immediate postoperative image of the donor site. Quilting sutures were done with negative suction drain placement in the donor site to prevent postoperation seromas.

**Figure 4:**
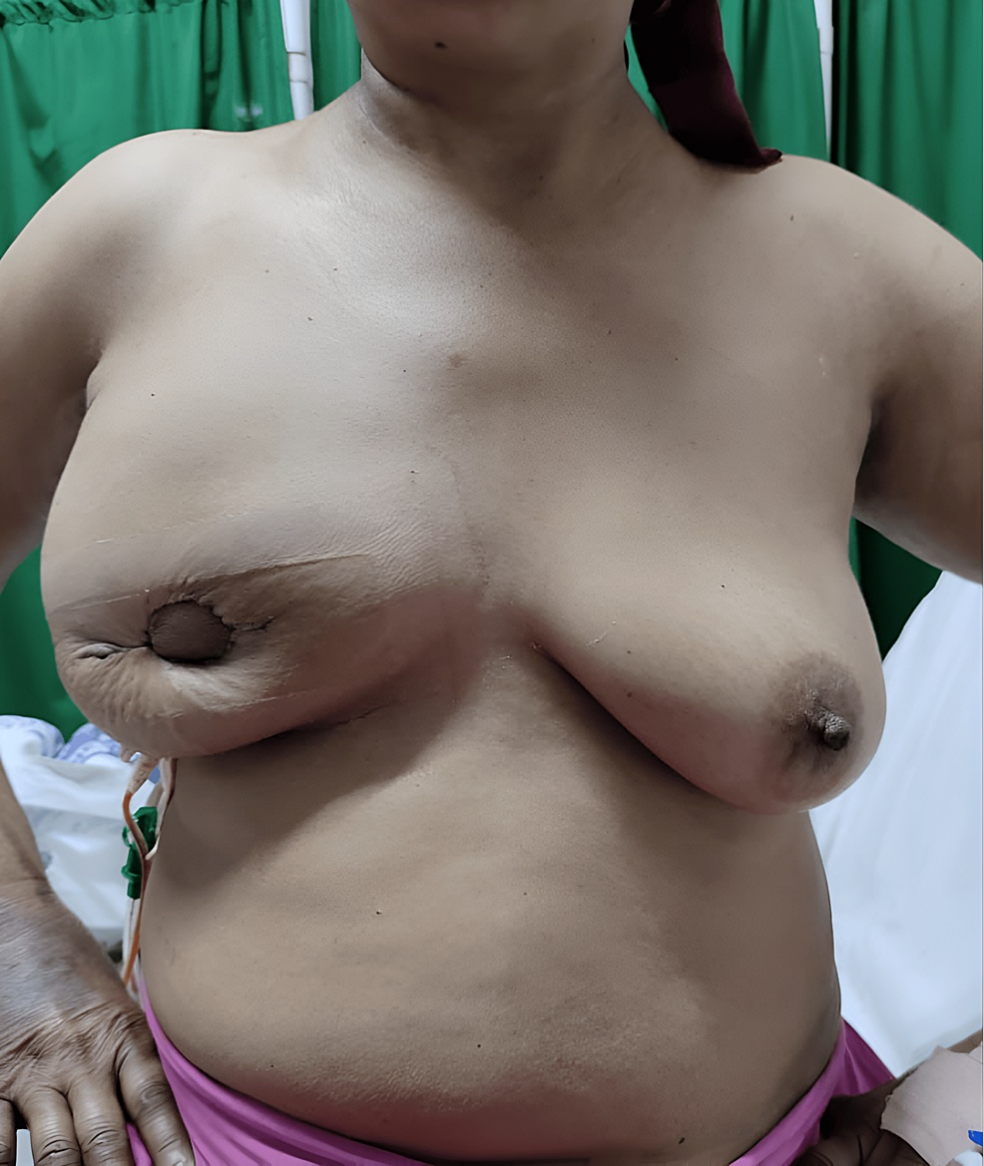
Postoperative day one (front view)

Digital photos were taken postoperatively from a fixed distance of 60 cm of the profile of bilateral breasts. Figure 4 shows a front view of the bilateral breasts on POD1.

**Figure 5:**
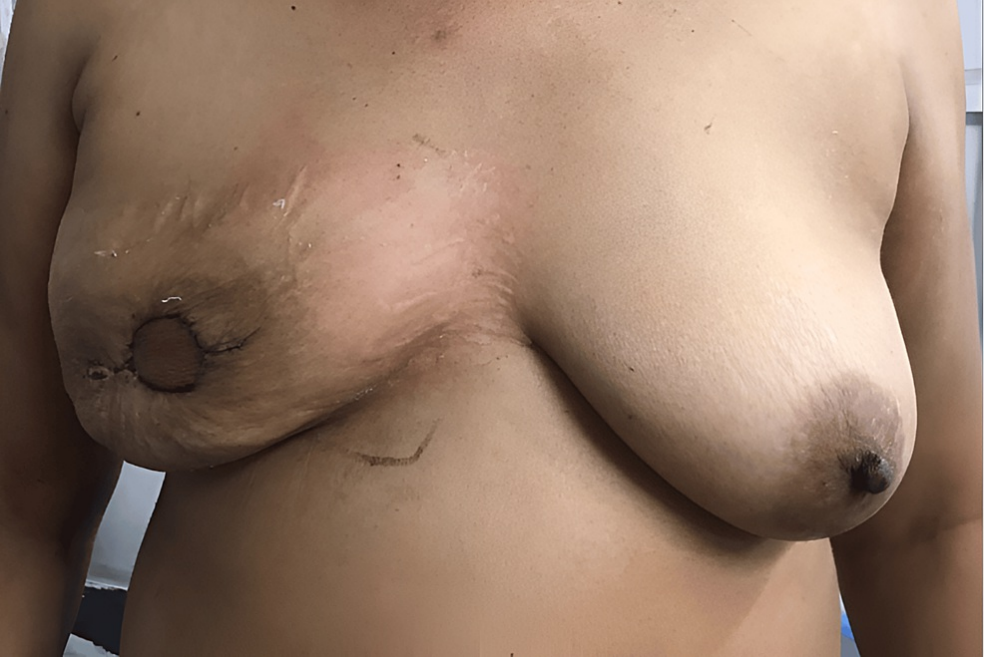
Bilateral breasts at postoperative week two

All patients were followed up at two weeks and six weeks postoperatively with digital photographs taken. Postoperative week two image of bilateral breasts is shown in Figure 5.

**Figure 6:**
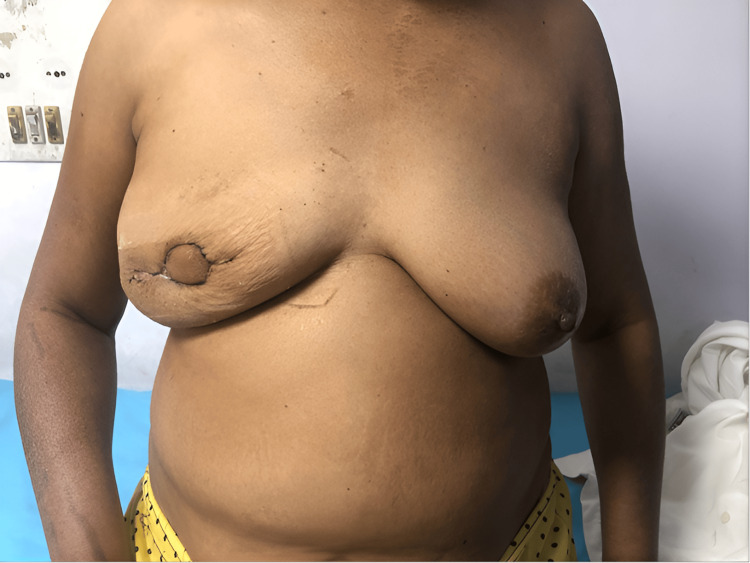
Six weeks postoperation

Figure 6 shows a postoperative week-six image showing the healed scar with a significant improvement in breast shape and contour.

## Discussion

In recent decades, oncoplastic breast surgery has shifted focus from radical mastectomy to breast preservation and reconstruction, prioritizing cosmetic outcomes alongside tumor removal [8]. There are several oncoplastic techniques that have been developed to meet the needs of different patients according to their tumor characteristics including the tumor size, tumor stage, and nodal status with the help of therapeutic volume reduction and correction of the defect using volume replacement [9,10], one of them being LD flap reconstruction as done in our study. However, in India, there’s limited documentation on oncological outcomes and cosmetic satisfaction following oncoplastic breast reconstruction, including procedures like LD flap reconstruction. Our study, involving 30 breast cancer patients, addresses this gap, aiming to assess both oncological outcomes and cosmetic satisfaction.

Our study group patients were similar to typical breast cancer surgery patients, with tumor characteristics consistent with the existing literature. Notably, patients were on average, a decade younger than expected. Reflecting national trends, a significant portion (n=14,46.7%) were aged 31-40 years, indicating a rising incidence of breast cancer in younger women. The National Cancer Registry Programme predicts a further increase in breast cancer cases, particularly among young women, by 2025 [2]. All patients in our study had a subjective assessment by the patients themselves on POD1, at week two, and at week six, while a double-blinded cosmetic assessment was done by a surgeon and nurse.

The patients were given BREAST-Q questionnaires, evaluating satisfaction with breasts, comfort wearing a brassiere, and impact on social and sexual lives, ensuring a comprehensive evaluation of psychosocial, sexual, and physical well-being. Statistical analysis revealed a dip in BREAST-Q scores on POD1 and week two compared to preoperative scores. However, by six weeks, postoperative scores had improved almost similar to preoperative scores. Objective assessments by the blinded surgeon and the blinded nurse also showed improvement at six weeks postoperation compared to two weeks postoperation. The improvement in the BREAST-Q score was due to the gradual healing of the surgical wound site over time, which resulted in a significant improvement in the breast shape and contour and a change in the patients’ perception of self-image and freedom from the psychological burden of the disease. Thus, the score improved from ‘somewhat satisfied’ to ‘very satisfied’ from POD1 to six weeks post operation. Our findings are consistent with previous research suggesting that patients undergoing oncoplastic breast surgery report higher satisfaction levels with their cosmetic outcomes rather than with mastectomy alone.

A reasonable part of the literature on the use of oncoplastic breast reconstructive surgery techniques over traditional mastectomies suggests better cosmetic outcomes with oncological resection. The LD flap reconstruction is a workhorse for reconstructive breast surgery procedures as reported by various studies. Li et al. [11] conducted a study at the Shanghai Fifth People’s Hospital, Fudan University, China, in 2021 and showed that patients who underwent LD myocutaneous flap after nipple and areola-sparing modified radical mastectomy (MRM) had reduced anxiety and depression with good aesthetic and oncological outcomes; this was similar to the positive impact on the quality of life of our patients. Sood et al. [12] also conducted a study in 2018 at the University of Miami, United States, and found the LD flap to be flexible and reliable in breast reconstruction, yielding satisfactory outcomes with acceptable short and long-term morbidities, consistent with our study findings. 

Escandón JM et al [13] found similar outcomes between obese and non-obese patients undergoing LD flap reconstruction in terms of surgical site complications and reliability and safe use of flap almost in parallel with our study group where none developed flap necrosis, and the complication rates were also acceptable. A study done by De Gournay et al. [14] found improved body image in LD flap reconstruction patients compared to mastectomy patients alone, consistent with our patients' satisfaction with body image after reconstruction. Kim et al. [15] also did a study that found comparable cosmetic outcomes between mini LD flap reconstruction and breast conservation surgery. While breast conservation surgery offered a better quality of life, a mini LD flap was a viable option for mastectomy patients desiring cosmetic satisfaction, particularly with large tumors. Our study aligns with the cited research, with most patients maintaining a mean BREAST-Q score of 79.2% at six weeks postoperation, similar to the preoperative score of 83.3%. This statistical analysis indicates that the satisfaction levels in quality of life are almost similar between the preoperative period and week six postoperation. Artificial intelligence has become a useful tool in assessing aesthetic outcomes and for regular follow-up of patients undergoing mastectomy for breast cancer [16]. 

Postoperative complications

In our study, five patients (16.7%) developed seromas and were managed by serial aspiration. A total of four patients (13.3%) had SSIs and were treated with adequate dressing; the SSIs healed over time. A few complained of shoulder weakness, which was resolved over time.

Limitations

In the Indian scenario, a lack of awareness of breast cancer among women often leads to late presentation and locally advanced breast cancer making breast reconstructive surgeries unfavorable. Thus, our study had a small sample size of only 30 patients who were fit for breast reconstructive surgery. Due to the short duration of the study, the assessment of the patients' quality of life post-radiation could not be taken for analysis. The questionnaires based on the patient-reported outcomes in the sexual well-being category could not be elicited properly due to social barriers and stigma faced by Indian women as opposed to the Western population. 

## Conclusions

Our study in comparison to various similar studies in terms of aesthetic and cosmetic assessment and quality of life had no significant difference between preoperative and six-week postoperative scores. There was also no significant difference between the objective scoring done by the blinded surgeon and the blinded nurse but a significant statistical difference was observed between the preoperative score and POD1 and week two scores (p-value <0.001). The mean age of the study population ranged between 31 and 50 years reflecting a rising trend of breast cancer in younger demographics, highlighting the importance of both cosmetic satisfaction and oncological outcomes. All patients were followed up for six weeks post operatively and were 100% disease-free with high oncological satisfaction and no distant metastasis.

Immediate LD flap breast reconstruction provides a reliable means for soft tissue coverage with acceptable perioperative morbidities for patients undergoing mastectomy. Complication rates were acceptable, with donor site seroma, SSI, and shoulder weakness among the complications. They could be prevented or treated (prolonged drain in situ, quilting sutures, and seroma aspiration) or resolved with time (SSI and shoulder function).
